# Refill Adherence Measures and Its Association with Economic, Clinical, and Humanistic Outcomes Among Pediatric Patients: A Systematic Review

**DOI:** 10.3390/ijerph17062133

**Published:** 2020-03-23

**Authors:** Brandon Chua, James Morgan, Kai Zhen Yap

**Affiliations:** 1 Department of Pharmacy, KK Women’s and Children’s Hospital, 100 Bukit Timah Road, Singapore 229899, Singapore; brandon_chua@live.com; 2 Department of Pharmacy, National University of Singapore, 18 Science Drive 4, Singapore 117543, Singapore; jmorgan812394@gmail.com

**Keywords:** clinical outcome, economic outcome, humanistic outcome, medication possession ratio, adherence measures, children

## Abstract

Although refill adherence measures (RAMs) are widely reviewed on their use among adult patients, existing reviews on adherence among children have only focused on self-report measures and electronic monitoring. Hence, this systematic review aims to examine the use of RAMs and their association with economic, clinical, and humanistic outcomes (ECHO) among pediatric patients. A literature search was conducted in Pubmed, Embase, CINAHL, and PsycINFO. Studies published in English involving subjects aged ≤18 years were included if RAMs were analyzed with ECHO. Of the 35 included studies, the majority (n = 33) were conducted in high-income countries. Asthma was the most common condition (n = 9) studied. Overall, 60.6% of 33 clinical outcomes reported among 22 studies was positive (improved clinical outcomes with improved adherence), while 21.9% of 32 economic outcomes reported among 16 studies was positive (reduced healthcare utilization or cost outcomes with improved adherence). Only four studies evaluated the relationship of adherence with 11 humanistic outcomes, where the majority (72.7%) were considered unclear. RAMs are associated with ECHO and can be considered for use in the pediatric population. Future studies could explore the use of RAMs in low-income countries, and the association of RAMs with quality of life.

## 1. Introduction

Medication nonadherence is associated with poor treatment outcomes and increased healthcare utilization among pediatric patients with chronic conditions [1,2]. However, the rate of medication nonadherence among these patients is estimated to be 50% [3,4]. An unnecessary increase in the burden on the healthcare system may result, as multiple urgent care and hospitalizations could be preventable with adequate medication adherence. Hence, measuring medication adherence is key to identifying patients at risk of poor treatment outcomes related to nonadherence and allowing for timely intervention strategies to be implemented.

Several objective and subjective measures of medication adherence have been developed, each with its own set of strengths and weaknesses [5,6]. However, the applicability of these measures remains a challenge among pediatric patients owing to age-related developments in pharmacokinetics, cognition, emotion, and social circumstances [1,2,7,8]. For example, measures that rely on self-reports are subjective and limited to patients with adequate cognitive ability to comprehend instructions. Although this can be circumvented by eliciting responses from caregivers, the overestimation of adherence may result due to negative pressures of reporting undesirable outcomes [5]. Direct objective measures, such as sampling of drug or metabolite levels, are invasive. Additionally, when working with the pediatric population, these samples require adjustments for age-related pharmacokinetics, in order to be clinically meaningful [9]. Electronic monitors have also been used as an indirect objective measure of real-time adherence behaviors [10]. However, the high cost involved with such technologies may limit its use in routine patient care.

In contrast, medication refill records provide an objective yet inexpensive source of information about medication adherence. Prescription refill patterns from insurance claims or individual pharmacy databases can be transformed into indicators to characterize medication adherence [9]. Common examples of such indicators or refill adherence measures (RAMs) include the medication possession ratio (MPR) and the proportion of days covered (PDC) [6]. These RAMs reflect the proportion of days of medication supply within a time interval and does not require additional input from patients or caregivers. Besides, population-level data can be analyzed concurrently, allowing for greater generalizability of findings [11]. However, since medication collection is assumed to correspond to consumption, the overestimation of adherence may result. Despite that, RAMs are commonly used in the adult population to explore the impact of medication adherence on health outcomes [12,13,14]. These outcomes can be broadly classified as economic, clinical, and humanistic outcomes (ECHO) [15], which allows for a comprehensive overview of the impacts of medication nonadherence. While self-reports and electronic monitoring have been extensively reviewed among pediatric patients [10,16,17], the use of RAMs in relation to health outcomes remains to be evaluated. Hence, the objective of this systematic review is to describe the use of RAMs among pediatric patients and examine its relationship with ECHO.

## 2. Materials and Methods

### 2.1. Search Strategy and Study Selection

The systematic review process was guided by the Preferred Reporting Items for Systematic Reviews and Meta-Analyses (PRISMA) statement [18]. A systematic search was done on PubMed, EMBASE, CINAHL, and PsycINFO to identify studies on the use of RAMs as a measure of adherence in the pediatric setting. The search strategy involved the keywords “adherence”, “pediatrics”, and “refill records” and their variant forms. The full search strategy is detailed in Table S1. The initial search was performed on 30 April 2019, and a final search was conducted on 13 February 2020 to maximize the currency of this review.

### 2.2. Inclusion and Exclusion Criteria

Studies were included if they were published in English, involved patients aged 18 years and below with chronic conditions, and analyzed ECHO with RAMs. Clinical outcomes included measures related to the treatment/control of disease or symptoms. Both healthcare utilization and cost were considered for economic outcomes. The patients’ and/or caregivers’ perspectives on functional health status, satisfaction, quality of life, and health-related knowledge were considered for humanistic outcomes. Hand-search of relevant studies were done on all review articles screened, and studies that met our inclusion criteria. Commentaries, editorials, case reports/series, dissertations, book chapters, and guidelines were excluded. Studies that involved both adult and pediatric patients were excluded if separate analyses were not reported for those aged ≤18 years old.

### 2.3. Data Extraction

Two independent reviewers performed the screening and extraction of the following data from the included studies: disease state, study setting, source of medication refill data, sample size, age of participants, definition of adequate adherence, ECHO. The ECHO were characterized as either positive, negative, mixed, or unclear based on their association with adherence. The positive category consisted of improved clinical or humanistic outcomes or reduced healthcare utilization/cost that were significantly associated with improved adherence. Conversely, outcomes were considered negative if increasing adherence was significantly associated with worse clinical or humanistic outcomes, or increased healthcare utilization/cost. Outcomes with no statistically significant association reported with adherence were regarded as unclear. Any outcomes with a combination of positive, negative, and unclear were considered as mixed. It has been highlighted that data from individual pharmacy databases are more sensitive to methodological differences in adherence calculations than claims databases, owing to a smaller dataset [19]. Hence, distinctions between data sources were made when the outcomes were reported.

### 2.4. Quality Assessment

The Newcastle–Ottawa Quality Assessment Scale (NOS) was used to ascertain the risk of bias of the included studies [20]. Similar to a study on pediatric medication adherence and healthcare utilization [1], only two fields in the NOS were used for quality assessment (representativeness of the cohort and outcome assessment method). Any disagreements between reviewers were resolved via discussion among the authors.

## 3. Results

**Figure 1:**
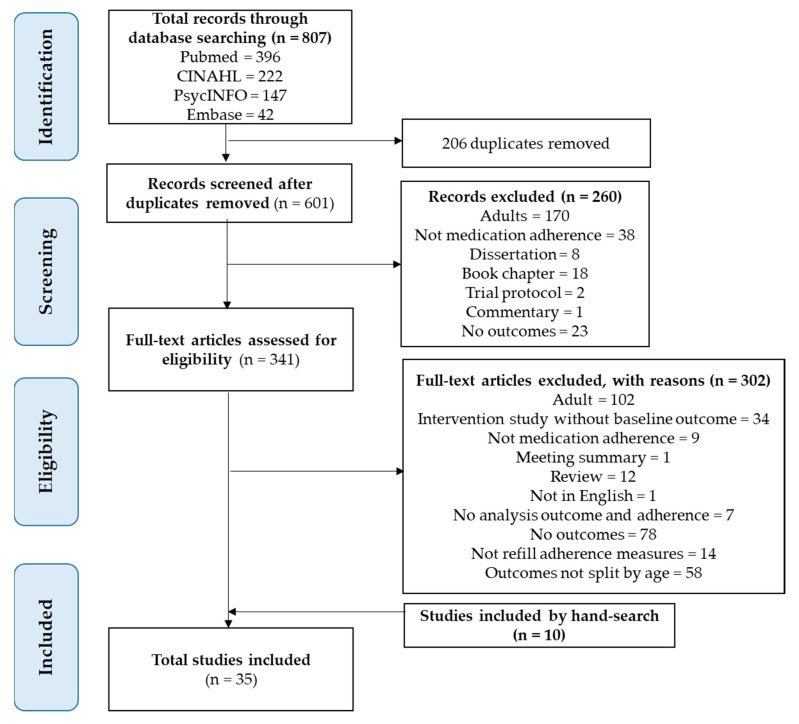
Preferred Reporting Items for Systematic Reviews and Meta-Analyses flow chart.

A total of 807 records were retrieved from the databases. After the removal of 206 duplicates and exclusion of another 260 records based on title and abstract, 341 studies were obtained for full-text screening. Another 302 studies were excluded upon full-text screening, and an additional 10 studies were identified from hand-search. Finally, a total of 35 articles were included for this review (Figure 1).

### 3.1. Study Characteristics

The characteristics of the included studies are described in Table 1. Of the 35 included studies, 26 were cohort studies [21,22,23,24,25,26,27,28,29,30,31,32,33,34,35,36,37,38,39,40,41,42,43,44,45,46], and 9 were cross-sectional studies [47,48,49,50,51,52,53,54,55]. The studies were conducted in 10 different countries, the majority (n = 33) of which were high-income countries, according to The World Bank definition [56]. These include the United States of America (n = 23) [21,22,23,24,25,26,27,29,31,32,33,34,35,39,41,47,48,49,50,51,52,53,54], United Kingdom (n = 3) [30,38,46], Israel (n = 2) [43,45], Taiwan (n = 1) [42], Korea (n = 1) [37], Australia (n = 1) [55], Netherlands (n = 1) [36], and Singapore (n = 1) [44]. Only two studies were conducted in upper–middle-income countries such as Brazil (n = 1) [28], and Malaysia (n = 1) [40]. No studies were conducted in lower middle-income and low-income countries. Asthma (n = 9) was the most common disease state involved in the studies [24,25,28,30,31,34,35,36,51], followed by human immunodeficiency virus (HIV) infection (n = 6) [21,22,23,47,49,50]. The sources of medication refill data were pharmacy records (n = 19) in approximately half of the studies, [21,22,23,28,33,35,37,40,44,46,47,48,49,50,51,52,53,54,55], and administrative claims database (n = 16) in the remaining studies [24,25,26,27,29,30,31,32,34,36,38,39,41,42,43,45]. In terms of the quality assessment using the NOS, 28 (80.0%) of all 35 studies involved a cohort that was deemed to be representative of the relevant pediatric population, and 24 (68.6%) studies used objective outcome measures (Table S2).

### 3.2. Measures of Adherence

The MPR and PDC were the most common RAMs reported (n = 19) [25,26,27,29,30,31,32,33,36,37,39,40,41,42,43,44,45,46,55]. The RAMs used in eight other studies were equivalent to the proportion of days with medication supply, similar to MPR and PDC [21,22,34,50,51,52,53,54]. Another four studies utilized RAMs involving the proportion of doses/refills collected in the study period [23,28,38,49], with ratio outputs comparable to MPR/PDC. The remaining four studies utilized the number of filled prescriptions as the RAMs [24,35,47,48].

Six studies did not define a level of adequate adherence for analysis with patient outcomes [23,28,29,32,51,55]. In one study, patients were divided into three adherence categories for analysis [26], while another study defined adequate adherence using a range of MPR between 0.80 to 1.20 (derived from prior studies) [30]. The remaining 29 studies defined adequate adherence with a fixed threshold below which patients were regarded as non-adherent. This ratio ranged from 0.08 in asthma [25], to 1.00 in HIV and type 1 diabetes (T1DM) [44,50]. Of the 27 studies with a fixed threshold to define adequate adherence, five studies derived its threshold from the study cohorts’ percentiles or data distributions [25,27,33,36,41], while 10 studies utilized thresholds from prior studies [22,31,37,39,40,43,45,49,50,53]. The source of threshold used for the remaining 12 studies were not specified [21,24,34,35,38,42,44,46,47,48,52,54].

### 3.3. Overview of Adherence and Patient Outcomes

**Figure 2:**
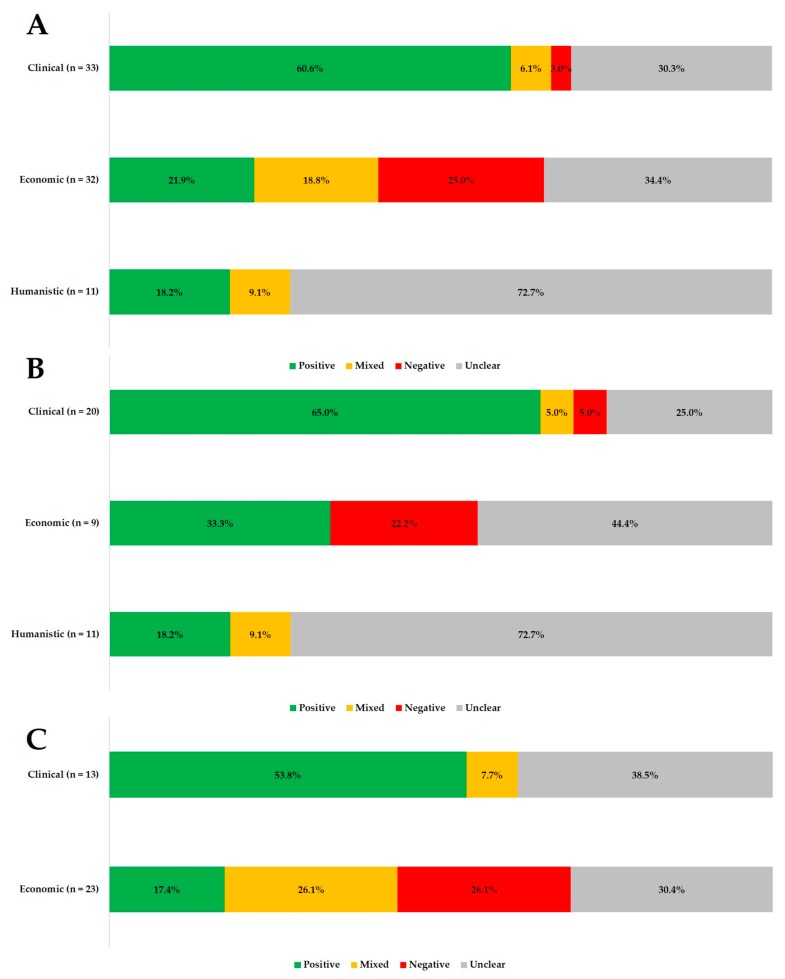
Categorization of economic, clinical, and humanistic outcomes in relation to medication adherence for studies that use (**a**) claims database and individual pharmacy database (**b**) individual pharmacy database only (**c**) claims database only. Positive: improved adherence significantly associated with improved clinical or humanistic outcome, or reduced healthcare utilization/cost. Negative: improved adherence significantly associated with reduced clinical or humanistic outcomes or improved healthcare utilization/cost. Unclear: no significant association reported between outcome and medication adherence. Mixed: a combination of positive, negative, and unclear.

Table S3 summarizes the reported relationship between medication adherence and ECHO. The majority of the studies (n = 22) reported the association of adherence to clinical outcomes, while 16 studies reported a relationship with economic outcomes. In contrast, only 4 studies reported humanistic outcomes. The majority (88.6%) of the studies assessed only one type of outcome, and no studies reported all ECHO. A total of 33 clinical outcomes, 32 economic outcomes, and 11 humanistic outcomes were reported in the 35 included studies. Figure 2 summarizes the categorization of these outcomes according to the association with medication adherence.

### 3.4. Adherence and Clinical Outcomes (n = 33)

When both sources of medication refill data were considered, a majority of the clinical outcomes (60.6%) had a positive association with adherence. Only one outcome in T1DM was considered negative, as the study reported a limited agreement between glycemic control and adherence [40]. The proportion of clinical outcomes categorized as positive was 65.0% among studies that utilized individual pharmacy data, compared to 53.8% among studies that utilized claims databases.

The 20 clinical outcomes among 14 studies that utilized individual pharmacy databases include blood pressure control in hypertension [33], fetal hemoglobin in sickle cell disease (SCD) [54], glycemic control in T1DM [40,44], vertical height in patients on growth hormones [46], and disease severity in cystic fibrosis (CF) [51]. Other outcomes were in HIV, such as viral load [21,22,23,47,49,50], and CD4 count [21]. Among patients with asthma, disease severity [35,51], lung function test [28,35], symptom control (exercise limitation, nocturnal symptom, morning symptoms) were assessed [28].

Eight studies that utilized claims database reported 13 clinical outcomes such as symptom control (activity limitation, nocturnal symptom, days with difficulty breathing) in chronic lung disease (CLD) [29], fracture risk due to furosemide in chronic heart disease [41], and seizure frequency in epilepsy [38]. Other clinical outcomes include disease remission and abnormal hematological parameters in inflammatory bowel disease (IBD) [39], graft failure and survival time in renal transplant [27], and positive bacteria cultures in rheumatic fever with penicillin prophylaxis [43]. Among patients with attention deficit disorder (ADD), all caused injury [26], and the risk of oppositional defiant disorder and conduct disorder were assessed [42].

### 3.5. Adherence and Economic Outcomes (n = 32)

The economic outcomes commonly involved the utilization of healthcare services and medication, while only one study involved the cost of treatment [31]. Adherence was not significantly associated with the majority (34.4%) of the economic outcomes. About 25% of the outcomes were categorized as negative, which reflected an increased healthcare resource with increasing adherence. This relationship was reported in six studies among patients with asthma, IBD, and those on mental health treatment [30,31,32,34,36,53]. A similar proportion of outcomes were classified as negative when studies using different sources of medication refill data were compared. In contrast, the proportion of economic outcomes categorized as positive was 33.3% among studies that utilized individual pharmacy data, compared to 17.4% among studies that utilized claims databases.

Utilization of healthcare services in the form of emergency visits, hospitalization, outpatient visits or contact with a healthcare worker, was commonly assessed among the six studies using individual pharmacy databases for patients with SCD [48], T1DM [44], asthma [28,35], IBD [53], and epilepsy [37]. One study among asthma patients also included systemic corticosteroid use among its utilization outcomes [28].

Among the 10 studies using claims database, similar health service utilization were reported for patients with out-of-home mental health treatment [32], asthma [24,25,31,34,36], CLD [29], and IBD [39]. In addition, medication utilization was assessed in CLD (antibiotics, systematic corticosteroid) [29], asthma (short-acting beta agonist, systematic corticosteroid) [30], and IBD (systematic corticosteroid and escalation of therapy) [39]. Patients on methylphenidate were also assessed for the future use of antidepressants [45]. The direct medical cost of asthma treatment was assessed in one study, which reported high costs among adherent patients compared to non-adherent patients [31].

### 3.6. Adherence and Humanistic Outcomes (n = 11)

In this systematic review, all 11 humanistic outcomes were reported by 4 studies that utilized individual pharmacy data. The majority (72.7%) of the outcomes were categorized as unclear, as no significant association with adherence was observed. These include patient knowledge of asthma [51], as well as caregiver perceived stress and knowledge in SCD [52]. Patient self-efficacy and knowledge of CF [51,55], and caregiver knowledge of CF [55], were also classified as unclear. Adherence was positively co-related with caregiver knowledge of medication in HIV [49], and caregiver knowledge of infection in SCD [52]. The relationship between adherence and patient knowledge of medication in CF differed by mediation type in one study and was classified as mixed [55]. No studies assessed the relationship between adherence and quality of life.

## 4. Discussion

This systematic review presents the first comprehensive summary of evidence from studies that measure pediatric medication adherence using RAMs. The value of RAMs as medication adherence measures is highlighted, especially when considering clinical outcomes. The majority of the clinical outcomes involved improvements in disease and symptom control with increasing adherence, even when different sources of medication refill data were considered. This relationship has also been reported in adult populations with chronic conditions using RAMs [57,58]. The direction of association between adherence and economic outcomes appears to be less distinct compared to clinical outcomes, as comparable proportions of “positive”, “negative”, “mixed”, and “unclear” relationships between adherence and outcomes are present in this review. Interestingly, a paradoxical relationship between improved adherence and higher healthcare resource use was also observed in studies among patients with asthma, IBD, and those on mental health treatment [30,31,32,34,36,53]. This could possibly be attributed to higher disease severity, where patients who are more ill may require closer follow up and incur greater medication expenditure. Hence, such possible influencing factors should be considered when analyzing the impacts of adherence to economic outcomes. In contrast, scant information is available for humanistic outcomes, with no studies assessing the quality of life. Therefore, more studies are required to assess the impact of adherence on humanistic outcomes, which can capture clinically relevant information that is important to patients.

Of the 35 studies included in this review, 16 used medication refill data from individual pharmacies. The outcomes of these studies were reported separately from those extracted from the other 19 studies using claims databases. Such a distinction is necessary, as the individual pharmacy datasets may not be complete, especially when patients can choose to fill their medications at different pharmacies. As a result, an underestimation of adherence may be observed. In addition, the datasets from individual pharmacies are comparatively smaller than administrative claims and are more sensitive to the effects of different methodologies employed for adherence calculations [19]. Although the larger datasets of claims database are ideal to overcome sample size issues common to pediatric-focused studies, it may be challenging and resource-intensive to gather data for analysis of humanistic outcomes with calculated adherence from claims database. This is because humanistic outcomes often rely on reports by patients or caregivers, which may not be routinely captured in administrative claims. Not surprisingly, none of the studies that reported humanistic outcomes in this review utilized claims databases. Hence, clear reporting of data source and adherence calculation is necessary, given that both sources of medication refill data have their own merits and limitation when used for adherence.

The use of RAMs appears to be limited in low-income countries. This was also reported in a review of pediatric adherence measures for HIV in low- and middle-income countries [59]. Although the use of RAMs has been regarded as relatively inexpensive, a centralized computerized system is often required for consistent inputs from prescribers and dispensers [6]. The availability of such infrastructures may be limited in low-income countries, such that the benefits of medication adherence monitoring may outweigh the manpower costs required to compute the indicators. Nevertheless, when available, it may outperform other measures of adherence, such as self-reports [60].

This review also identified a wide variation in the definition of adequate adherence among studies with RAMs that is expressed as a ratio such as MPR and PDC. This ranged from 0.08 to 1.00 depending on the disease state of interest, which calls for caution when comparing adherence rates across disease states. Ten studies used pre-defined thresholds from prior research studies, while only five studies derived the adherence threshold from their studies’ cohort percentiles or data distributions. Often, a threshold of 0.80 has been regarded as adequate adherence and has been applied in policy as a quality measure [61]. However, this threshold can differ by disease state [62], patient outcome of interest [63], and patient’s health status [64], within the same study cohort. In light of these factors, a cohort-specific threshold should be considered in relation to targeted outcomes when conducting adherence studies, and this should be compared with threshold published in the literature in sensitivity analyses [65].

The use of RAMs has their own inherent limitations, especially since actual consumption of medications cannot be determined. In addition, distinctions between intentional nonadherence, which is deliberate, and unintentional adherence cannot be made. However, these limitations may be mitigated with the concurrent use of self-reported adherence in a composite adherence measure [66]. Information from both adherence measures can be “triangulated” to provide a more accurate assessment of adherence behaviors [10,67]. In the absence of a gold-standard adherence measure, such approaches for composite adherence measures have been suggested to more accurately capture the information required to determine adherence [6]. Besides that, this study has to be interpreted in light of the following limitations. A number of studies (n = 58) were excluded as patient outcomes or adherence rates were not reported separately for pediatric and adult patients. This could have introduced selection bias of the studies included. Nevertheless, owing to different factors affecting pediatric and adult medication adherence, these studies were excluded. In addition, the results cannot be generalized to the entire pediatric patient population as several major pediatric chronic conditions such as juvenile idiopathic arthritis and cancer have not been represented in this review. Lastly, the small cohort sizes in some disease states such as SCD, hypertension, and CF may limit the generalizability of the results within the same disease state. Hence, more studies are warranted in these disease states to further guide the use of RAMs as a measure of medication adherence.

## 5. Conclusions

Similar to adult populations, RAMs can be considered to characterize medication-taking behavior among pediatric patients. Despite the limitations of RAMs as an adherence measure, this review has demonstrated evidence of the association between RAMs and ECHO, especially with clinical outcomes. However, information on the relationship between medication adherence and humanistic outcomes is limited to self-efficacy, stress, and health-related knowledge, with none covering quality of life. Given the wide variation in the definition of adequate adherence, challenges remain when interpreting studies using RAMs. While several major pediatric diseases have been studied, few studies have explored the use of RAMs in low-income countries. Therefore, further studies are also warranted to explore the impacts of RAMs on quality of life, as well as its feasibility in patient populations from low-income countries.

## Figures and Tables

**Table 1:** Characteristics of the included studies.

Author (year)	Study Setting	Medication Refill Data Source	Study Design (Duration/ Time Scale)	Medication Refill Indicator/ Definition of Adequate Level of Adherence	Sample Size	Study Participants’ Age (Years)
**Asthma**						
Elkout et al. (2012)	UK	Claims database	Retrospective cohort study (5 years)	MPR: 0.80–1.20	3172	Mean ICS only: 6 LI: 6 LABA/ICS: 9 LABA + ICS: 8
Herndon et al. (2012)	USA	Claims database	Retrospective cohort study (3 years)	MPR ≥ 0.50	18,456	Range (number of patients) 2–4: (4203) 5–11: (9998) 12–18: (4255)
Camargo et al. (2007)	USA	Claims database	Retrospective cohort study (4.5 years)	BIS: MPR > 0.08 Non-nebulized ICS: MPR >0.08 LI: MPR > 0.16	10,976	Mean (SD) 3.8 (2.2)
Lasmar et al. (2009)	Brazil	Individual pharmacy	Prospective cohort study (1 year)	Adherence rate^1^: no specific threshold defined	122	Median 6 (range: 3.1–12.2)
Bickel et al. (2016)	USA	Individual pharmacy	Prospective cohort study (6 months)	First prescription filled within 14 days	77	Mean 6.35 (4.58)
Engelkes et al. (2016)	Netherlands	Claims database	Retrospective cohort study (12 years)	MPR > 0.87	14,303	10.2 (4.0)
Bukstein et al. (2007)	USA	Claims database	Retrospective cohort study (9 years)	Number of prescriptions filled: ≥ 2 post-index date	11,407	Mean (SD) 2.2 (0.92)
Rust et al. (2013)	USA	Claims database	Retrospective cohort study (1 year)	Proportion of prescribed days covered^2^ ≥ 0.50	43,156	Mean (SD) Controller-to-total asthma medication ratio ≥ 0.5: 8.1 (2.4) Controller-to-total asthma medication ratio < 0.5: 7.8 (2.3)
**Human immunodeficiency virus infection**						
Watson et al. (1999)	USA	Individual pharmacy	Retrospective cohort study (6 months)	Pharmacy refill rate^3^ ≥ 0.75	72	Range (number of patients) 3–23 months: 11 2–5 years: 28 6–12 year: 33
Marhefka et al. (2004)	USA	Individual pharmacy	Prospective cross-sectional study (3 months)	Pharmacy refill rate ^1^ ≥ 0.90	51	Mean age (SD) 8.76 (3.06)
Burack et al. (2010)	USA	Individual pharmacy	Prospective cross-sectional study (3 years)	Number of prescriptions filled: No missing refill in 6 months	46	Mean (SD) Viral load ≤ 400 copies/ml: 11.2 (3.4) Viral load > 400 copies/ml: 11.5 (3.1)
Marhefka et al. (2006)	USA	Individual pharmacy	Prospective cross-sectional study (not specified)	Pharmacy refill rate^3^ = 1.00	51	Mean (range) 8 (2–12)
Farley et al. (2003)	USA	Individual pharmacy	Prospective cohort study (2 years)	Pharmacy refill rate^1^: no specific threshold defined	26	Mean (SD) 6.9 (3.2)
Katko et al. (2001)	USA	Individual pharmacy	Prospective cohort study (1 year)	Pharmacy refill rate^3^ ≥ 0.90	34	Median (range) Adherent: 7.5 (1.50–16.3) Non-adherent: 8.9 (2.90–19.9)
**Attention deficit disorder**						
Wang et al. (2018)	Taiwan	Claims database	Retrospective cohort study (12 years)	MPR ≥ 0.50	ODD cohort: 32168CD cohort: 32676	Mean (SD) ODD cohort: 9.13 (2.87) CD cohort: 9.15 (2.86)
Marcus et al. (2008)	USA	Claims database	Retrospective cohort study (4 years)	Low: MPR < 0.30 Medium: MPR 0.30–0.70 High: MPR > 0.70	11,770	Range (number of patients) 6–12: 9,916 13–17: 1854
**Inflammatory bowel disease**						
Oliva-Hemker et al. (2007)	USA	Individual pharmacy	Prospective cross-sectional study (2 years)	Refill score^3^ ≥ 0.80	51	Mean 14.2 (3.2)
Samson et al. (2017)	USA	Claims database	Retrospective cohort study (1 year)	MPR ≥ 0.80	228	Mean (SD) MPR < 0.80: 16.7 (3.3) MPR ≥ 0.80: 15.7 (3.3)
**Sickle cell disease**						
Thornburg et al. (2010)	USA	Individual pharmacy	Prospective cross-sectional study (3 years)	Duration of supply: ≥ 5 months in 6 months period	75	Mean (range) 11.2 (3.5–17.8)
Witherspoon et al. (2006)	USA	Individual pharmacy	Prospective cross-sectional study (not defined)	Duration without medication: ≤ 7 days per month	30	Mean (SD) 2.95 (1.48)
Elliot et al. (2001)	USA	Individual pharmacy	Prospective cross-sectional study (5 months)	Prescription filled within 14 days	50	Mean month (SD) 28.3 (16.0)
**Epilepsy**						
Lee et al. (2016)	Korea	Individual pharmacy	Retrospective cohort study (3.5 years)	MPR ≥ 0.80	1172	Range (number of patients): 1 (51) 2–5 (208) 6–11 (486) 12–18 (427)
Shetty et al. (2016)	UK	Claims database	Retrospective cohort study (2 years)	Adherence Index^1^ > 0.90	320	Median (IQR) 10 (7–14)
**Congenital heart disease**						
Heo et al. (2018)	USA	Claims database	Retrospective cohort study(7 years)	MPR ≥ 0.70	Propensity score matched cohort: 3912	Mean age (SD) MPR ≥ 0.70: 1.67 (2.30) MPR < 0.70: 1.51 (2.06) Not on furosemide: 1.58 (2.08)
**Type 1 diabetes mellitus**						
Ying et al. (2017)	Malaysia	Individual pharmacy	Retrospective cohort study (5 years)	MPR ≥ 0.80	57	Mean (SD) 14.4 (3.41)
Chua et al. (2019)	Singapore	Individual pharmacy	Retrospective cohort study (5 years)	MPR ≥ 1.00	206	Mean (SD) Adherent: 11.6 (3.7) Non-adherent: 12.4 (4.1)
**Renal transplant**						
Chisholm-Burns et al. (2009)	USA	Claims database	Retrospective cohort study (6 years)	MPR ≥ 0.92	877	Mean (SD) 11.9 (5.35)
**Hypertension**						
Eakin et al. (2013)	USA	Individual pharmacy	Prospective cohort study (not specified)	MPR ≥ 0.65	21	Mean (SD) 14.7 (2.0)
**Chronic lung disease**						
Collaco et al. (2010)	USA	Claims database	Prospective cohort study (2.5 years)	MPR: no specific threshold defined	194 of which 33 had prescription claims	Mean month (SD) Patients with prescription claims: - At discharge: 4.3 (2.5) - At first clinic visit: 7.0 (3.1)
**Patients receiving psychiatric residential/foster care**						
Robst et al. (2012)	USA	Claims database	Retrospective cohort study (3 years)	MPR: no specific threshold defined	2304 treatment episodes	Range (number of patients) 6–12: 749 13–17: 1,555
**Patients on methylphenidate**						
Madjar et al. (2019)	Israel	Claims database	Prospective cohort study (12 years)	MPR ≥ 0.50	6834	Non-ADM, age, (number of patients): 6: 1555 7: 2295 8: 2414 ADM, age, (number of patients): 6: 156 7: 227 8: 187
**Cystic fibrosis**						
Faint et al. (2017)	Australia	Individual pharmacy	Prospective cohort study (6 months)	MPR: no specific threshold defined	39	Median 14 (range: 12–17)
**Cystic fibrosis or asthma**						
Modi et al. (2006)	USA	Individual pharmacy	Prospective cross-section study (3 months)	Prescription refill rate^3^: no specific threshold defined	73	Mean Cystic fibrosis: 10.1 Asthma: 9.7
**Patients on growth hormones**						
Michaelidou et al. (2019)	UK	Individual pharmacy	Retrospective cohort study (3 years)	PDC > 0.80	52	Mean (SD) 8.50 (3.78)
**Rheumatic fever**						
Amarilyo et al. (2019)	Israel	Claims database	Retrospective cohort study (19 years 5 months)	PDC > 0.80	842	Mean (SD) Oral: 8.6 (3.7) Intramuscular: 10.9 (3.2)

ADM, antidepressant medication; BIS, budesonide inhalation suspension; CD, conduct disorder; ICS, inhaled corticosteroid; LABA, long-acting beta 2 agonist; LI, leukotriene inhibitor; MPR, medication possession ratio; ODD, oppositional defiant disorder; PDC, proportion of days covered. ^1^ Adherence rate/adherence index/compliance rate/pharmacy refill rate= $\frac{Doses\;refilled\;in\;follow\;up\;period}{Doses\;prescribed\;in\;follow\;up\;period}$ ^2^ Proportion of prescribed days covered = $\frac{Days\;of\;supply\;prescribed\;in\;follow\;up\;period}{Days\;in\;follow\;up\;period}$ ^3^ Prescription refill rate/pharmacy refill rate/medication refill adherence/refill score = $\frac{Days\;of\;supply\;obtained\;in\;follow\;up\;period}{Days\;in\;follow\;up\;period}$

## Supplementary Materials

The following are available online at https://www.mdpi.com/1660-4601/17/6/2133/s1, Table S1: Search strategy for systematic review. Table S2: Quality assessment of included studies using the Newcastle–Ottawa scale. Table S3: Association of medication adherence with clinical, economic, and humanistic outcomes.
