# Supplementary material for: Refill Adherence Measures and Its Association with Economic, Clinical, and Humanistic Outcomes Among Pediatric Patients: A Systematic Review
Source: Int J Environ Res Public Health. 2020 Mar 23;17(6):2133. doi: 10.3390/ijerph17062133 (PMC7142643; doi:10.3390/ijerph17062133)
Supplement: Supplementary file 1 [file ijerph-17-02133-s001.pdf]

Table S1. search strategy according to databases.

Pubmed		
**No.**	**Search terms**	**Hits**
1	(“medication possession ratio” OR “proportion of days covered” OR “continuous multiple-interval of medication availability” OR “continuous multiple interval of medication availability” OR “continuous multiple-interval of medication gap” OR “continuous multiple interval of medication gap” OR “continuous multiple-interval measure of over-supply” OR “continuous multiple interval measure of over-supply” OR “continuous multiple-interval measure of oversupply” OR “continuous multiple interval measure of oversupply” OR “continuous single-interval of medication availability” OR “continuous single interval of medication availability” OR “continuous single-interval of medication gap” OR “continuous single interval of medication gap” OR “days between fills” OR “medication refill” OR “proportion of prescribed days covered” OR “pharmacy refill” OR “pharmacy refills” OR “pharmacy claim” OR “pharmacy claims” OR “claims database” OR “claim database” OR “medication claim” OR “medication claims” OR “pharmacy administrative data” OR “pharmacy record data” OR “pharmacy administrative claim” OR “pharmacy administrative claims” OR “pharmacy record claim” OR “pharmacy record claims” OR “pharmacy database” OR “pharmacy record” OR “pharmacy records” OR “pharmacy dispensing” OR “dispensing database” OR “medication database” OR “medicine database” OR “medication record” OR “medication records” OR “medicine record” OR “medicine records” OR “medication refills” OR "medicine refill” OR “medicine refills” OR “refill” OR “refills”)	12584
2	(“adherence” OR “non-adherence” OR “nonadherence” OR “compliance” OR “non-compliance” OR “noncompliance” OR “concordance” OR “non-concordance” OR “nonconcordance” OR “adherent” OR “compliant” OR “adhered” OR “adhering” OR “adhere” OR “complied” OR “comply” OR “complying” OR “concord” OR “concorded” “concording” OR “nonadherent” OR “non-adherent” OR “noncompliant” OR “non-compliant” OR “nonconcordant” OR “non-concordant” OR “nonadhering” OR “non-adhering” OR “noncomplying” OR “non-complying” OR “nonconcording” OR “non-concording” OR Medication Adherence[Mesh])	30190
3	(“pediatrics” OR “pediatric” OR “adolescent” OR “infant” OR “juvenile” OR “kid” OR “kids” OR “teenager” OR “toddler” OR “youth” OR “neonate” OR “neonates” OR “neonatal” OR “preteen” OR “preteens” OR “pre-teen” OR “pre-teens” OR “child” OR “teen” OR “teens” OR “children” OR “newborn” OR “pubescent” OR “prepubescent” OR “pre-pubescent” OR “paediatric” OR “paediatrics” OR “adolescents” OR “teenagers” OR “youths” OR “adolescence” OR “preschool” OR “pre-school” OR “childhood” OR “infancy” OR Child[Mesh] OR infant[Mesh] OR Adolescent[Mesh])	4475882
4	1 AND 2 AND 3	410
5	4 AND filter: Human	404
7	6 AND filter: English	396

Embase		
**No.**	**Search terms**	**Hits**
1	(‘medication possession ratio’ OR ‘proportion of days covered’ OR ‘continuous multiple-interval of medication availability’ OR ‘continuous multiple interval of medication availability’ OR ‘continuous multiple-interval of medication gap’ OR ‘continuous multiple interval of medication gap’ OR ‘continuous multiple-interval measure of over-supply’ OR ‘continuous multiple interval measure of over-supply’ OR ‘continuous multiple-interval measure of oversupply’ OR ‘continuous multiple interval measure of oversupply’ OR ‘continuous single-interval of medication availability’ OR ‘continuous single interval of medication availability’ OR ‘continuous single-interval of medication gap’ OR ‘continuous single interval of medication gap’ OR ‘days between fills’ OR ‘medication refill’ OR ‘proportion of prescribed days covered’ OR ‘pharmacy refill’ OR	25336

	‘pharmacy refills’ OR ‘pharmacy claim’ OR ‘pharmacy claims’ OR ‘claims database’ OR ‘claim database’ OR ‘medication claim’ OR ‘medication claims’ OR ‘pharmacy administrative data’ OR ‘pharmacy record data’ OR ‘pharmacy administrative claim’ OR ‘pharmacy administrative claims’ OR ‘pharmacy record claim’ OR ‘pharmacy record claims’ OR ‘pharmacy database’ OR ‘pharmacy record’ OR ‘pharmacy records’ OR ‘pharmacy dispensing’ OR ‘dispensing database’ OR ‘medication database’ OR ‘medicine database’ OR ‘medication record’ OR ‘medication records’ OR ‘medicine record’ OR ‘medicine records’ OR ‘medication refills’ OR ‘medicine refill’ OR ‘medicine refills’ OR ‘refill’ OR ‘refills’)	
2	(‘adherence‘ OR ‘non-adherence’ OR ‘nonadherence’ OR ‘compliance’ OR ‘non-compliance’ OR ‘noncompliance’ OR ‘concordance’ OR ‘non-concordance’ OR ‘nonconcordance’ OR ‘adherent’ OR ‘compliant’ OR ‘adhered’ OR ‘adhering’ OR ‘adhere’ OR ‘complied’ OR ‘comply’ OR ‘complying’ OR ‘concord’ OR ‘concorded’ ‘concording’ OR ‘nonadherent’ OR ‘non-adherent’ OR ‘noncompliant’ OR ‘non-compliant’ OR ‘nonconcordant’ OR ‘non-concordant’ OR ‘nonadhering’ OR ‘non-adhering’ OR ‘noncomplying’ OR ‘non-complying’ OR ‘nonconcording’ OR ‘non-concording’)	20649
3	(‘pediatrics’ OR ‘pediatric’ OR ‘adolescent’ OR ‘infant’ OR ‘juvenile’ OR ‘kid’ OR ‘kids’ OR ‘teenager’ OR ‘toddler’ OR ‘youth’ OR ‘neonate’ OR ‘neonates’ OR ‘neonatal’ OR ‘preteen’ OR ‘preteens’ OR ‘pre-teen’ OR ‘pre-teens’ OR ‘child’ OR ‘teen’ OR ‘teens’ OR ‘children’ OR ‘newborn’ OR ‘pubescent’ OR ‘prepubescent’ OR ‘pre-pubescent’ OR ‘paediatric’ OR ‘paediatrics’ OR ‘adolescents’ OR ‘teenagers’ OR ‘youths’ OR ‘adolescence’ OR ‘preschool’ OR ‘pre-school’ OR ‘childhood’ OR ‘infancy’)	5202437
4	1 AND 2 AND 3	94
5	4 AND ([article]/lim OR [article in press]/lim OR [review]/lim) AND ([newborn]/lim OR [infant]/lim OR [child]/lim OR [preschool]/lim OR [school]/lim OR [adolescent]/lim) AND [humans]/lim AND [english]/lim<br><br>filter: article, article in press, review, newborn, infant, child, preschool, school, adolescent, human, English	42

**CINAHL**		
**No.**	**Search terms**	**Hits**
1	(“medication possession ratio” OR “proportion of days covered” OR “continuous multiple-interval of medication availability” OR “continuous multiple interval of medication availability” OR “continuous multiple-interval of medication gap” OR “continuous multiple interval of medication gap” OR “continuous multiple-interval measure of over-supply” OR “continuous multiple interval measure of over-supply” OR “continuous multiple-interval measure of oversupply” OR “continuous multiple interval measure of oversupply” OR “continuous single-interval of medication availability” OR “continuous single interval of medication availability” OR “continuous single-interval of medication gap” OR “continuous single interval of medication gap” OR “days between fills” OR “medication refill” OR “proportion of prescribed days covered” OR “pharmacy refill” OR “pharmacy refills” OR “pharmacy claim” OR “pharmacy claims” OR “claims database” OR “claim database” OR “medication claim” OR “medication claims” OR “pharmacy administrative data” OR “pharmacy record data” OR “pharmacy administrative claim” OR “pharmacy administrative claims” OR “pharmacy record claim” OR “pharmacy record claims” OR “pharmacy database” OR “pharmacy record” OR “pharmacy records” OR “pharmacy dispensing” OR “dispensing database” OR “medication database” OR “medicine database” OR “medication record” OR “medication records” OR “medicine record” OR “medicine records” OR “medication refills” OR "medicine refill” OR “medicine refills” OR “refill” OR “refills”)	4961
2	(“adherence ” OR “non-adherence” OR “nonadherence” OR “compliance” OR “non-compliance” OR “noncompliance” OR “concordance” OR “non-concordance” OR “nonconcordance” OR “adherent” OR “compliant” OR “adhered” OR “adhering” OR “adhere” OR “complied” OR “comply”	138667

	OR “complying” OR “concord” OR “concorded” “concording” OR “nonadherent” OR “non-adherent” OR “noncompliant” OR “non-compliant” OR “nonconcordant” OR “non-concordant” OR “nonadhering” OR “non-adhering” OR “noncomplying” OR “non-complying” OR “nonconcording” OR “non-concording”)	
3	(“pediatrics” OR “pediatric” OR “adolescent” OR “infant” OR “juvenile” OR “kid” OR “kids” OR “teenager” OR “toddler” OR “youth” OR “neonate” OR “neonates” OR “neonatal” OR “preteen” OR “preteens” OR “pre-teen” OR “pre-teens” OR “child” OR “teen” OR “teens” OR “children” OR “newborn” OR “pubescent” OR “prepubescent” OR “pre-pubescent” OR “paediatric” OR “paediatrics” OR “adolescents” OR “teenagers” OR “youths” OR “adolescence” OR “preschool” OR “pre-school” OR “childhood” OR “infancy”)	1115965
4	1 AND 2 AND 3	281
5	4 AND English Language; Human; Age Groups: Infant, Newborn: birth-1 month, Infant: 1-23 months, Child, Preschool: 2-5 years, Child: 6-12 years, Adolescent: 13-18 years	222

**PsychINFO**		
**No.**	**Search terms**	**Hits**
1	medication possession ratio.mp.	206
2	proportion of days covered.mp.	112
3	continuous multiple-interval of medication availability.mp.	0
4	continuous multiple interval of medication availability.mp.	0
5	continuous multiple-interval of medication gap.mp.	0
6	continuous multiple interval of medication gap.mp.	0
7	continuous multiple-interval measure of over-supply.mp.	0
8	continuous multiple interval measure of over-supply.mp.	0
9	continuous multiple-interval measure of oversupply.mp.	2
10	continuous multiple interval measure of oversupply.mp.	2
11	continuous single-interval of medication availability.mp.	0
12	continuous single interval of medication availability.mp.	0
13	continuous single-interval of medication gap.mp.	0
14	continuous single interval of medication gap.mp.	0
15	days between fills.mp.	0
16	medication refill.mp.	31
17	pharmacy refill.mp.	69
18	pharmacy refills.mp.	12
19	pharmacy claim.mp.	17
20	pharmacy claims.mp.	225
21	claims database.mp.	392
22	claim database.mp.	12
23	medication claim.mp.	1
24	medication claims.mp.	6
25	pharmacy administrative data.mp.	0
26	pharmacy record data.mp.	1
27	pharmacy administrative claim.mp.	0
28	pharmacy administrative claims.mp.	0
29	pharmacy record claim.mp.	0
30	pharmacy record claims.mp.	0
31	pharmacy database.mp.	51
32	pharmacy record.mp.	7
33	pharmacy records.mp.	174
34	pharmacy dispensing.mp.	52
35	dispensing database.mp.	4
36	medication database.mp.	2
37	medicine database.mp.	46

38	medication record.mp.	13
39	medication records.mp.	40
40	medicine record.mp.	1
41	medicine records.mp.	1
42	medication refill.mp.	31
43	medication refills.mp.	40
44	medicine refill.mp.	0
45	medicine refills.mp.	0
46	refill.mp.	357
47	refills.mp.	241
48	1 or 2 or 3 or 4 or 5 or 6 or 7 or 8 or 9 or 10 or 11 or 12 or 13 or 14 or 15 or 16 or 17 or 18 or 19 or 20 or 21 or 22 or 23 or 24 or 25 or 26 or 27 or 28 or 29 or 30 or 31 or 32 or 33 or 34 or 35 or 36 or 37 or 38 or 39 or 40 or 41 or 42 or 43 or 44 or 45 or 46 or 47	1714
49	(adherence or nonadherence or compliance or noncompliance or concordance or nonconcordance or adherent or compliant or adhered or adhering or adhere or complied or comply or complying or concord or concorded or concording or nonadherent or noncompliant or nonconcordant or nonadhering or noncomplying or nonconcording).mp.	75317
50	non-adherence.mp.	1689
51	non-compliance.mp.	1192
52	non-concordance.mp.	17
53	non-adherent.mp.	481
54	non-compliant.mp.	378
55	non-concordant.mp.	26
56	non-adhering.mp.	2
57	non-complying.mp.	2
58	non-concording.mp.	0
59	exp Compliance/ or exp Treatment Compliance/	19189
60	49 or 50 or 51 or 52 or 53 or 54 or 55 or 56 or 57 or 58 or 59	75332
61	(pediatrics or pediatric or adolescent or infant or juvenile or kid or kids or teenager or toddler or youth or neonate or neonates or neonatal or preteen or preteens or child or teen or teens or children or newborn or pubescent or prepubescent or pre-pubescent or paediatric or paediatrics or adolescents or teenagers or youths or adolescence or preschool or childhood or infancy).mp. [mp=title, abstract, heading word, table of contents, key concepts, original title, tests & measures]	1108567
62	pre-teen.mp.	98
63	pre-teens.mp.	59
64	pre-school.mp.	3385
65	PEDIATRICS/ or CHRONICALLY ILL CHILDREN/	24849
66	61 or 62 or 63 or 64 or 65 or 66	1108708
66	48 and 60 and 66	147

Table S2. Quality assessment using the Newcastle-Ottawa Scale.

Author, year	Study design (time scale/duration)	Representativeness of the exposed cohort	Assessment of outcome
Marcus, et al (2008)	Retrospective cohort study	✓	✓
Wang, et al (2018)	Retrospective cohort study	✓	✓
Camargo, et al (2007)	Retrospective cohort study	✓	✓
Bukstein, et al (2007)	Retrospective cohort study	✓	✓
Lasmar, et al (2009)	Prospective cohort study	X	✓
Herndon, et al (2012)	Retrospective cohort study	✓	✓
Elkout, et al (2012)	Retrospective cohort study	✓	✓
Rust, et al (2013)	Retrospective cohort study	✓	✓
Bickel, et al (2016)	Prospective cohort study	X	✓
Engelkes, et al (2016)	Retrospective cohort study	✓	✓
Modi, et al (2006)	Prospective cross-section study	✓	X
Faint, et al (2017)	Prospective cross-sectional study	✓	X
Heo, et al (2018)	Retrospective cohort study	✓	✓
Collaco, et al (2010)	Prospective cohort study	✓	X
Shetty, et al (2016)	Retrospective cohort study	✓	✓
Lee, et al (2016)	Retrospective cohort study	✓	✓
Watson, et al (1999)	Retrospective cohort study	✓	✓
Farley, et al. (2003)	Prospective cohort study	✓	X
Marhefka, et al (2004)	Prospective cross-sectional study	✓	✓
Marhefka, et al (2006)	Prospective cross-sectional study	✓	X
Burack, et al (2010)	Prospective cross-sectional study	X	X
Eakin, et al (2013)	Prospective cohort study	X	X
Oliva-Hemker, et al (2007)	Prospective cross-sectional study	✓	✓
Samson, et al (2017)	Retrospective cohort study	✓	✓
Madjar, et al (2019)	Prospective cohort study	✓	✓
Robst, et al (2012)	Retrospective cohort study	✓	✓
Elliot, et al (2001)	Prospective cross-sectional study	X	X

Witherspoon, et al (2006)	Prospective cross-sectional study	X	X
Thornburg, et al (2010)	Prospective cross-sectional study	✓	X
Chong, et al (2017)	Retrospective cohort study	✓	✓
Chisholm-Burns, et al (2009)	Retrospective cohort study	✓	✓
Michaelidou, et al (2019)	Prospective cross-sectional study	✓	✓
Amarilyo, et al (2019)	Retrospective cohort study	✓	✓
Katko, et al (2001)	Prospective cohort study	X	X
Chua, et al (2019)	Retrospective cohort study	✓	✓

Supplementary Table S3: Association of medication adherence with clinical, economic and humanistic outcomes

Author (year)	Relationship between adherence and patient outcomes		
	Clinical	Economic	Humanistic
**Asthma**			
Elkout, et al (2012)	-	• **≥6 SABA canisters prescribed per year (mixed)**<br>- 89% to 285% higher odds of adherent patients on ICS, LI, LABA/ICS (MPR 0.80 to 1.20) prescribed with SABA compared to non-adherent patents (MPR<0.80 or MPR>1.20)<br>- No significant association between adherence and SABA use among patients on LABA+ICS<br><br>• **Systemic corticosteroid prescribed in the post index year (unclear)**<br>- No significant association	-
Herndon, et al (2012)	-	• **Emergency department visit (mixed)**<br>- 32 to 44% lower odds of emergency visits for patients on ICS and LI with MPR≥0.50 compared to MPR 0.00 to 0.19<br>- No significant association for patients on ICS and LI with MPR 0.20 to 0.49 compared to MPR 0.00 to 0.19<br>`<br>• **Hospitalization (mixed)**<br>- 27% higher odds of hospitalization for patients on ICS with MPR: 0.20 - 0.49 compared to MPR: 0 - 0.19<br>- No significant association for patients on ICS and LI with MPR≥0.50 compared to MPR 0.00 to 0.19<br>- No significant association for patients on LI with MPR 0.20 to 0.49 compared to MPR 0.00 to 0.19<br><br>• **Total asthma care cost (negative)**<br>- Cost incurred by patients on ICS and LI with MPR≥0.50 was three times as high compared to those with MPR 0.00 to 0.19<br><br>• **Predicted overall expenditure (negative)**<br>- Cost incurred by patients on ICS and LI with MPR≥0.50 was three times as high compared to those with MPR 0.00 to 0.19	**-**
Camargo, et al (2007)	-	• **Hospitalization or emergency department visit (positive)**<br>- 68% to 75% lower odds for hospitalization or emergency visits for adherent patients on BIS (MPR>0.08), non-nebulized ICS (MPR>0.08) and LI (MPR>0.16)	-

Lasmar, et al (2009)	• **Morning symptoms (positive)**<br>- Higher median adherence rate among patients without symptoms (values unspecified)<br><br>• **Nocturnal symptoms (positive)**<br>- Higher median adherence rate among patients without symptoms (values unspecified)<br><br>• **Exercise limitation (positive)**<br>- Higher median adherence rate among patients without limitation (values unspecified)<br><br>• **$FEV_1 \geq 80\%$ (positive)**<br>- Higher median adherence rate among patients $FEV_1 \geq 80\%$ (values unspecified)	• **Asthma exacerbation (treated with systemic corticosteroids or emergency department visit or hospitalization) (positive)**<br>- Higher median adherence rate for patients with no exacerbation (values unspecified)	-
Bickel, et al (2016)	• **$FEV_1 \geq 80\%$ (unclear)**<br>- No significant association<br><br>• **Disease severity (unclear)**<br>- No significant association	• **Returned for follow-up appointment (unclear)**<br>- No significant association<br>•	-
Engelkes, et al (2016)	-	• **Specialist visits (mixed)**<br>- Lower proportion of patients with MPR>0.87 (6%) have ≥1 visit compared to those with MPR<0.37 (15%)<br>- Patients with MPR>0.87 have 50% higher rate of visits per patient-year compared to those with MPR<0.37<br><br>• **Exacerbation (hospitalizations, emergency department visit or corticosteroid use) (mixed)**<br>- Lower proportion of patients with MPR>0.87 (4%) exacerbated compared to those with MPR<0.37 (7%)<br>- Patients with MPR>0.87 have 21% higher rate of exacerbations per patient-year compared to those with MPR<0.37	**-**

Bukstein, et al (2007)	-	• **Hospitalization or emergency department visit (mixed)**<br>- No significant association between adherence (all asthma controller medications) and hospitalization or emergency visits<br>- Patients on nebulized ICS with ≥2 prescriptions post-index period had 40% lower odds of hospitalization or emergency visits compared to those with <2 prescriptions	-
Rust, et al (2013)	-	• **Emergency department visit (negative)**<br>- Patients with PPDC<0.50 had 7% lower odds of admission compared to those with PPDC≥0.50<br><br>• **Hospitalization (negative)**<br>- Patients with PPDC<0.50 had 38% lower odds of admission compared to those with PPDC≥0.50	-
**Human immunodeficiency virus infection**			
Watson, et al (1999)	• **Viral load (positive)**<br>Adherent patients (pharmacy refill rate≥0.75) had 9.90 times lower odds of achieving and maintaining viral load<400 copies/ml compared to non-adherent patients (pharmacy refill rate<0.75)<br><br>• **CD4 count (positive)**<br>- Adherent patients (pharmacy refill rate≥0.75) had a higher increase in counts (10% vs <5%) at 240 days of therapy compared to non-adherent patients (pharmacy refill rate<0.75)	-	-
Marhefka, et al (2004)	• **Viral load (mixed)**<br>- Significant association when adherence analysed as refill rate≥0.90 compared to refill rate<0.90<br>- No significant association when adherence analysed as a continuous variable	-	• **Caregiver knowledge of medication (accurate identification of prescribed medications) (positive)**<br>- Moderate correlation with adherence (r= 0.34)
Burack, et al (2010)	• **Viral load (unclear)**<br>- No significant association	-	-
Marhefka, et al (2006)	• **Viral load (positive)**	-	-

	- Limited agreement with adherence (kappa= -0.26)		
Farley, et al. (2003)	• **Viral load (positive)**<br>- Inversely related with adherence (adjusted R^2= 0.39)	-	-
Katko, et al. (2001)	• **Viral load (positive)**<br>- Higher proportion of patients pharmacy refill rate>0.90 (50%) with have virologic response compared to those with pharmacy refill<0.90 (13%)	-	-

Attention deficit disorder

Wang, et al (2018)	• **Risk of developing ODD (positive)**<br>- 53% lower odds among patients with MPR≥0.50 compared to those MPR<0.50<br><br>• **Risk of developing CD (positive)**<br>- 58% lower odds among patients with MPR≥0.50 compared to MPR<0.50	-	-
Marcus, et al (2008)	• **All-cause injury rate (unclear)**<br>- No significant association	-	-

Sickle cell disease

Thornburg, et al (2010)	• **Fetal hemoglobin (positive)**<br>- Moderate correlation with adherence (r=0.39)	-	-
Witherspoon, et al (2006)	-	-	• **Caregiver PSS (unclear)**<br>- No significant association<br><br>• **Caregiver knowledge of infection score (positive)**<br>- Moderate correlation with adherence (r= 0.56)<br><br>• **Caregiver knowledge of death score (unclear)**<br>- No significant association
Elliot, et al (2001)	-	• **Hospitalization (positive)**<br>- Moderate correlation with adherence (r = -0.38)	-

Epilepsy

Lee, et al (2016)	-	• **Hospitalization or emergency department visits (positive)**<br>- Patients with MPR<0.80 had 2.1 times higher risk of admissions compared to those with MPR≥0.80	-
Shetty, et al (2016)	• **Seizure frequency (unclear)**	-	-

	- No significant association		

Inflammatory bowel disease

Oliva-Hemker, et al (2007)	-	• **Emergency department visit (negative)**<br>- Patients with refill score≥0.80 had more visits (0.80) compared to patients with refill score<0.80 (0.17)<br><br>• **Number of healthcare contacts (negative)**<br>- Patients with refill score≥0.80 had higher number of contacts (6.1) compared to patients with refill score<0.80 (3.0)	-
Samson, et al (2017)	• **Disease remission (positive)**<br>- Higher mean MPR among patients in remission (0.72 vs 0.51)<br>- Patients with MPR≥0.80 had 7 times higher odds of remission defined by PGA compared to patients with MPR<0.80<br>- Patients with MPR≥0.80 had 3 times higher odds of remission defined by disease activity score compared to patients with MPR<0.80<br><br>• **Abnormal hematological parameters (erythrocyte sedimentation rate, C-reactive protein, hemoglobin, hematocrit) (unclear)**<br>- No significant association	• **Escalation of therapy (positive)**<br>- Higher proportion of patients with MPR<0.80 (25%) required escalation compared with those with MPR≥0.80 (3%)<br><br>• **Hospitalization (unclear)**<br>- No significant association<br><br>• **Repeat course of corticosteroid (unclear)**<br>- No significant association	-

Cystic fibrosis

Faint, et al (2017)	-	-	• **General Self-Efficacy Scale (unclear)**<br>- No significant association<br><br>• **Patient Knowledge of Disease Management-CF score (mixed)**<br>- Significant correlation with adherence to Hypertonic saline (R^2 = 0.40)<br>- No significant association with adherence to dornase alfa and multivitamins<br><br>• **Caregiver Knowledge of Disease Management-CF score (unclear)**<br>- No significant association
**Congenital heart disease**			
Heo, et al (2018)	• **Fractures (mixed)**<br>- 87% higher odds among patients with MPR≥0.70 compared against patients not on furosemide<br>- 54% higher odds patients with MPR<0.70 compared against patients not on furosemide<br>- Shorter time to fracture among patients with MPR≥0.70 and MPR<0.70 compared to patients not on frusemide<br>- No significant difference between time to fracture between patients with MPR≥0.70 and MPR<0.70	-	-
**Type 1 diabetes**			
Ying, et al (2017)	• **HbA1c (negative)**<br>- Limited agreement with adherence (kappa=-0.108)	-	-
Chua, et al (2019)	• **HbA1c (positive)**<br>**-** lower mean HbA1c among patients with MPR=1.00 compared with patients with MPR<1.00	• **Emergency department visit (unclear)**<br>- No significant association<br>`<br>• **Hospitalization (unclear)**<br>- No significant association<br><br>• **Outpatient visit (unclear)**<br>- No significant association	-
**Renal transplant**			
Chisholm-Burns et al (2009)	• **Graft failure (positive)**<br>- Patients with MPR≤0.92 was 2.1 times as likely to experience graft failure compared to patients with MPR>0.92<br>- 8% reduced risk for every 0.1 increase in MPR	-	-<br>-

	• **Graft survival time (positive)**<br>- Patients with MPR>0.92 had longer graft survival times compared to those with MPR≤0.92 (values unspecified)		
**Hypertension**			
Eakin, et al (2013)	• **Blood pressure control (positive)**<br>- Higher proportion of patients with MPR≥0.65 (100%) attained control compared to those with MPR<0.65 (36%)	-	-
**Chronic lung disease**			
Collaco, et al (2010)	• **Activity limitation (positive)**<br>- Every 0.1 increase in MPR associated with 29% lower odds of activity limitation<br><br>• **Days with difficulty breathing (unclear)**<br>- No significant association<br><br>• **Night time symptoms (unclear)**<br>- No significant association	• **Emergency visit (positive)**<br>- Every 0.1 increase in MPR associated with 25% lower odds of emergency visit<br><br>• **Rescue medication use (positive)**<br>- Every 0.1 increase in MPR associated with 16% lower odds of rescue medication use<br><br>• **Hospitalization (unclear)**<br>- No significant association<br><br>• **Steroid use (unclear)**<br>- No significant association<br><br>• **Antibiotic use (unclear)**<br>- No significant association	-
**Rheumatic fever**			
Amarilyo, et al (2019)	• **Positive group A Streptococcus cultures (positive)**<br>- Lower proportion of positive cultures among patients with PDC>0.80 (2.5%) compared to those with PDC≤0.80 (17.4%)	-	-
**Patients receiving psychiatric residential/foster care**			
Robst, et al (2012)	-	• **Contact with mental health practitioners, physicians and case managers (negative)**<br>- Higher MPR among youth with more healthcare contacts<br><br>• **Returning to psychiatric residential/foster care in 6 months (unclear)**<br>- No significant association	-
**Patients on methylphenidate**			
Madjar, et al (2019)	-	• **Prescribed with antidepressant during adolescence (negative)**<br>- Patients with MPR≥0.50 had 50% increased odds of antidepressants prescribed compared to those with MPR<0.50	-
**Patients on growth hormone**			

Michaelidou, et al (2019)	• **Height standard deviation score (positive)**<br>- Year-on-year increment over 3 years among patients with PDC>0.80 but not those from PDC≤0.80<br>- Increase from baseline after 3 years of treatment among patients with PDC>0.80 only but not those from PDC≤0.80	-	-
**Patients with asthma or CF**			
Modi, et al (2006)	• **Asthma disease severity (unclear)**<br>- No significant association<br>• **Cystic fibrosis disease severity (unclear)**<br>- No significant association	-	• **Caregiver asthma knowledge (unclear)**<br>- No significant association<br><br>• **Caregiver cystic fibrosis knowledge (unclear)**<br>- No significant association<br><br>• **Patient asthma knowledge (unclear)**<br>- No significant association<br><br>• **Patient cystic fibrosis knowledge (unclear)**<br>- No significant association

BIS, Budesonide inhalation suspension; CD4, Cluster of differentiation 4; CD, Conduct disorder; FEV_1: forced expiratory volume in 1 second; ICS, Inhaled corticosteroid; LI, Leukotriene inhibitors; MPR, Medication possession ratio; MSTAQ, Multiple sclerosis treatment adherence questionnaire; ODD, Oppositional defiant disorder; PGA, Physician global assessment; PSS, Perceived Stress Scale; PDC, Proportion of days covered; Proportion of prescribed days covered, PPDC; SABA, Short-acting beta-agonist.

Categorization of outcomes: Positive: improved adherence significantly associated with improved clinical or humanistic outcome, or reduced healthcare utilization/cost. Negative: improved adherence significantly associated with reduced clinical or humanistic outcome or improved healthcare utilization/cost. Unclear: no significant association reported between outcome and medication adherence. Mixed: combination of positive, negative and unclear.

BIS, Budesonide inhalation suspension; CD4, Cluster of differentiation 4; CD, Conduct disorder; FEV1: forced expiratory volume in 1 second; ICS, Inhaled corticosteroid; LI, Leukotriene inhibitors; MPR, Medication possession ratio; MSTAQ, Multiple sclerosis treatment adherence questionnaire; ODD, Oppositional defiant disorder; PGA, Physician global assessment; PSS, Perceived Stress Scale; PDC, Proportion of days covered; Proportion of prescribed days covered, PPDC; SABA, Short-acting beta-agonist

Categorization of outcomes: Positive: improved adherence significantly associated with improved clinical or humanistic outcome, or reduced healthcare utilization/cost. Negative: improved adherence significantly associated with reduced clinical or humanistic outcome or improved healthcare utilization/cost. Unclear: no significant association reported between outcome and medication adherence. Mixed: combination of positive, negative and unclear
